# A Prospective Clinical Study of Ferric Citrate Hydrate for Chronic Heart Failure with Iron Deficiency Anemia

**DOI:** 10.3390/life15040598

**Published:** 2025-04-03

**Authors:** Akira Sezai, Hisakuni Sekino, Makoto Taoka, Kazuaki Obata, Sakie Kanno, Masashi Tanaka

**Affiliations:** 1 Department of Cardiovascular Surgery, Nihon University School of Medicine, Tokyo 173-8610, Japan; taoka.makoto@nihon-u.ac.jp (M.T.); tanaka.masashi@nihon-u.ac.jp (M.T.); 2 Sekino Hospital, Tokyo 171-0014, Japan; sekinoh@sekino-hospital.com (H.S.); obatak@sekino-hospital.com (K.O.); s.kanno.mt@gmail.com (S.K.)

**Keywords:** iron-deficiency anemia, anemia, heart failure, ferric citrate hydrate, chronic heart failure

## Abstract

Background: The efficacy of intravenous iron preparations for chronic heart failure with iron deficiency has been reported, but the efficacy of oral iron preparations has not been demonstrated. In this study, we conducted a prospective clinical study using ferric citrate hydrate tablets in patients with chronic heart failure complicated by iron deficiency anemia. Methods and Results: A prospective study was conducted using ferric citrate hydrate in patients with chronic heart failure complicated by iron deficiency anemia. The registered patients were divided into two groups: those administered ferric citrate hydrate and those switched from iron sulfate sustained-release to ferric citrate hydrate. The primary endpoint was hemoglobin level. The secondary endpoints included hematocrit, serum iron, saturation, ferritin, and cardiac-, renal-, and hepatic-related biomarkers. A total of 141 patients were enrolled in this study, including 95 patients who were newly administered ferric citrate hydrate and 46 patients who were switched from iron sulfate sustained-release to ferric citrate hydrate. Conclusions: Ferric citrate hydrate significantly increased hemoglobin, serum iron, transferrin saturation (TSAT), and ferritin levels, and decreased atrial natriuretic peptide (ANP), brain natriuretic peptide (BNP), and N-terminal pro-brain natriuretic peptide (NT-proBNP) levels. Ferric citrate hydrate could be continued without side effects such as gastrointestinal symptoms. Improvement in iron metabolism and anemia due to iron supplementation with ferric citrate hydrate led to improvement in heart failure biomarkers.

## 1. Introduction

The combination of heart failure and anemia is common, with anemia (hemoglobin (Hb) < or = 12.0 g/dL) present in 12% of the 912 patients with chronic heart failure enrolled in the RENAISSANCE trial. For every 1 g/dL higher baseline Hb, a 15.8% reduction in risk of mortality and a 14.2% reduction in risk of death or hospitalization for heart failure were reported. In addition, more severe chronic heart failure was associated with significantly lower Hb levels and was a significant and independent predictor of death or heart failure hospitalization [1]. Reports from Japan indicate that anemia was present in 60% of patients with acute decompensated heart failure [2,3] and in 35% of patients with chronic heart failure (C). In these reports, anemia is also considered an independent prognostic factor for heart failure patients [2,4].

The causes of anemia in heart failure include (1) hemofiltration due to fluid retention, (2) decreased erythropoietin production due to chronic kidney disease, (3) decreased bone marrow hematopoietic function due to inflammatory cytokine activity stimulation, (4) iron deficiency, etc. [5], but there is no established evidence on how to treat anemia. Furthermore, it has also been reported that heart failure patients often have iron deficiency [6,7]. The cause is a disturbance in iron utilization due to hepcidin, which is thought to play an important role in anemia and iron metabolism in heart failure [8,9]. Hepcidin is mainly produced by hepatocytes, and excess hepcidin causes iron deficiency and anemia by inhibiting iron absorption from the intestine and inhibiting the release of iron from macrophage stores [10]. Inflammation is also involved, and it induces hepcidin expression via interleukin-6 [11].

Clinical studies of iron administration for heart failure with iron deficiency are being conducted regardless of whether or not the patient has anemia. There was reported that iron deficiency was found in 62% of 7160 patients diagnosed with chronic heart failure, while anemia, according to WHO criteria, was found in 35% of these patients [12]. Intravenous iron preparations for patients with chronic heart failure have been reported to improve symptoms, increase the distance walked in six minutes, and reduce the number of hospitalizations due to worsening heart failure, regardless of whether the patient has anemia or not [13,14], and intravenous iron preparations are recommended for heart failure with iron deficiency in the European Society of Cardiology guidelines [15]. However, no results have been shown to indicate the effectiveness of oral iron supplements. In the IRONOUT HF study, which was a trial of oral iron preparations, an iron polysaccharide formulation was used, and it was hoped that it would be possible to ingest the most elemental iron of any commercially available oral supplement and that its tolerability profile would help with medication compliance and minimize the risk of subjects being unblinded, but it was not possible to demonstrate efficacy [16]. In addition, most of the clinical research has been on chronic heart failure with iron deficiency, and there has been little clinical research on chronic heart failure with iron deficiency anemia.

In Japan, there are currently five types of oral iron deficiency anemia medication: Ssodium ferrous citrate tablets, ferric fumarate capsules, iron sulfate sustained-release tablets, soluble ferric pyrophosphate syrup, and ferric citrate hydrate tablets. In this study, a prospective clinical study was conducted using ferric citrate hydrate tablets in patients with chronic heart failure complicated by iron deficiency anemia, and the enrolled patients were divided into two groups: those who were newly administered ferric citrate hydrate tablets and those who were switched from iron sulfate sustained-release tablets (Fero-Gradumet Tablets) to ferric citrate hydrate tablets.

## 2. Materials and Methods

### 2.1. Study Protocol

The subjects of this study were patients with chronic heart failure and iron deficiency anemia. The definition of chronic heart failure in this study was cases with a history of heart failure hospitalization and taking two or more standard heart failure medications (angiotensin II receptor blockers (ARBs), angiotensin-converting enzyme (ACE) inhibitors, angiotensin receptor neprilysin inhibitors (ARNIs), mineralocorticoid receptor antagonists (MRAs), beta blockers, diuretics, or inotropic agents). Furthermore, the subjects were limited to patients whose prescriptions, including those for heart failure medications, had remained unchanged for at least 6 months. In this study, we did not specify the left ventricular ejection fraction.

The definition of iron deficiency anemia was an Hb level of less than 11 g/dL for men and less than 10.5 g/dL for women, and a ferritin level of less than 100 ng/mL or a ferritin level of 100 to 299 ng/mL with a TSAT of less than 20%. In addition, patients were defined as having a negative occult blood test and having other types of anemia ruled out by measuring vitamin B12, lactate dehydrogenase (LDH), and haptoglobin. Although this is an open-label study, assessors were blinded.

The age range of eligible patients was ≥20 years to <100 years. The exclusion criteria were (1) patients who did not meet the definition of chronic heart failure and iron deficiency anemia specified in the inclusion criteria, (2) patients who were judged by the investigator to be unable to take oral medications properly due to dementia or other reasons, (3) patients with unstable heart failure, (4) pregnant women, and (5) women who were breastfeeding. Finally, patients who were judged by the investigator to be unsuitable for this study were excluded. 

Patients were assigned to oral treatment with ferric citrate hydrate (Riona^®^, 500 mg/once/day after dinner, Torii Pharmaceutical Co., Ltd., Tokyo, Japan). Ferric citrate hydrate is a drug covered by insurance since 2021 to treat iron deficiency anemia. When the Hb level was 13 g/dL or higher after administration of ferric citrate hydrate, the dose was reduced to 250 mg; if the level was still 13 g/dL or higher after that, the drug was discontinued. The extraction and statistical processing of the test data was carried out by a third party not involved in this study. Blood samples were taken in the morning. The iron content of the ferric citrate hydrate tablets used in this study was 124 mg per 500 mg, and that of the iron sulfate sustained-release tablets (Fero-Gradumet^®^, 105 mg/one time/day, Viatris Inc., Tokyo, Japan) was 105 mg.

The study details were explained to each patient, and informed consent was obtained. The study was approved by the hospital’s institutional review board (protocol no. 20210401 and approval on 1 June 2021), and the study was registered with the Hospital Medical Information Network (study ID: UMIN000050436).

The primary endpoint was the Hb level. The secondary endpoints were as follows: New York Heart Association (NYHA) classification; hematocrit (Ht); serum iron; TSAT = (Fe/total iron binding capacity (TIBC)) × 100; ferritin; cardiac-related biomarkers (ANP, BNP, NT-proBNP); renal-related biomarkers (blood urea nitrogen (BUN), creatinine (Cr), estimated glomerular filtration rate (eGFR)); hepatic-related biomarkers (aspartate aminotransferase (AST), alanine aminotransferase (ALT)); oxidized low-density lipoprotein (Ox-LDL); and high-sensitivity C-reactive protein (hs-CRP).

The definition of MACCE in this study included death, ischemic heart disease, cerebrovascular disease, heart failure, and arrhythmia requiring hospitalization for treatment.

All blood samples were measured before administration. ANP, BNP, NT-proBNP, and Ox-LDL were measured at 3 and 6 months after administration, while the other parameters were measured at 1, 3, and 6 months after administration.

Adverse reactions were classified as renal dysfunction (increased Cr by 50%), hepatic dysfunction (increased AST/ALT by ≥50%), and allergic reactions. The attending physician decided the management of the reactions.

### 2.2. Statistical Analysis

Measured values were expressed as the mean standard deviation (SD). A *p* value of less than 0.05 was considered statistically significant. Each dataset was analyzed using repeated measures ANOVA, and Bonferroni’s method was used to adjust for multiple comparisons. NYHA classification data were analyzed using Student’s *t*-test. All analyses were performed with SPSS software (SPSS Inc., Chicago, IL, USA, version number: 28.0.0.0, copyright holder: IBM SPSS Statistics). Data aggregation was performed by Sekino Laboratory staff who were not involved in this study, and statistical analysis was supported by Data Seed Inc. (Tokyo, Japan). Data Seed Inc. was not involved in conducting the study.

## 3. Results

A total of 141 patients were enrolled in this study, with 95 patients receiving ferric citrate hydrate for the first time and 46 patients switching from iron sulfate sustained-release tablets to ferric citrate hydrate. Among the newly administered cases, there were seven where the patient had taken iron supplements in the past and was unable to continue taking them due to digestive symptoms.

The baseline characteristics are shown in Table 1. Regarding classifications of heart failure in this study, in the new administration cases, 25% had heart failure with reduced ejection fraction (HFrEF), 21% had heart failure with mildly reduced ejection fraction (HFmrEF), and 54% had heart failure with preserved ejection fraction (HFpEF). In the switching cases, 35% had HFrEF, 19% had HFmrEF, and 46% had HFpEF, so about half of the cases were HFpEF. In terms of age, there were 33 (35%) and 71 (74%) cases of new administration in patients aged 80 and over, and 25 (54%) and 38 (83%) cases of switching in patients aged 80 and over. Regarding CKD, there were 48 cases (51%) of new administration cases with CKD stage G3a or below, 40 cases (42%) with stage G3b or below, and 12 cases (13%) with stage G4 or below. For the switching group, there were 26 cases (57%) with CKD stage G3a or below, 24 cases (52%) with stage G3b or below, and eight cases (17%) with stage G4 or below. There were 77 (81%) in the new treatment group and 11 (24%) in the switching group. Regarding ferritin levels of less than 100 ng/mL, there were 90 (95%) in the new treatment group and 24 (52%) in the switching group.

### Adverse Events

There were no readmissions due to heart failure or cardiovascular events during the 6-month period of this study. In the new administration group, there were no patients who were unable to continue taking ferric citrate hydrate due to gastrointestinal symptoms, but in the switching group, there was one case of nausea and one case of diarrhea. The patient who experienced diarrhea discontinued the drug after 2 months of administration. The patient who discontinued treatment was excluded from the primary analysis.

**Figure 1:**
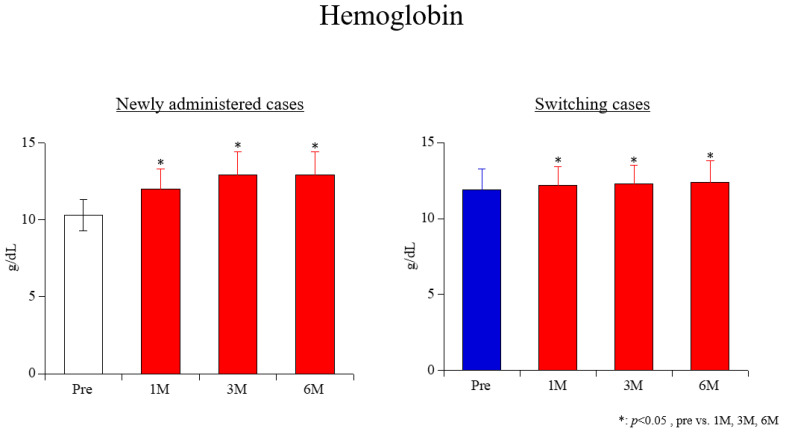
Changes in hemoglobin levels.

- <Newly administered cases:>
- Primary endpoint:

Hb (Figure 1): Before the administration of ferric citrate hydrate, the hemoglobin level was 10.3 ± 1.0 g/dL. After 1 month of treatment, it increased to 12.0 ± 1.3 g/dL; after 3 months, it was 12.9 ± 1.5 g/dL; and after 6 months, it remained at 12.9 ± 1.5 g/dL. These values show a statistically significant increase compared to baseline (all; *p* < 0.001). Furthermore, hemoglobin levels at 3 and 6 months were significantly higher than those at 1 month (*p* < 0.001 for both comparisons).

- Secondary endpoints:

NYHA classification (Table 2): No patients experienced worsening of NYHA classification following ferric citrate hydrate treatment. Compared to baseline, NYHA classification improved significantly after treatment (*p* < 0.001).

Ht, Fe, TSAT, and ferritin (Table 2): Ht, serum iron, TSAT, and ferritin levels all increased significantly after ferric citrate hydrate administration compared to baseline (all *p* < 0.001). Ht increased significantly at 3 and 6 months compared to 1 month (both *p* < 0.001). Serum iron levels were significantly higher at 6 months than at 3 months (*p* = 0.014). TSAT did not differ significantly across the treatment periods (1 vs. 3 months: *p* = 0.109; 1 vs. 6 months: *p* = 0.964; 3 vs. 6 months: *p* = 0.056). Ferritin levels increased significantly at 3 and 6 months compared to 1 month (1 vs. 3 months: *p* = 0.002; 1 vs. 6 months: *p* < 0.001), and levels at 6 months were also significantly higher than at 3 months (*p* < 0.001).

**Figure 2:**
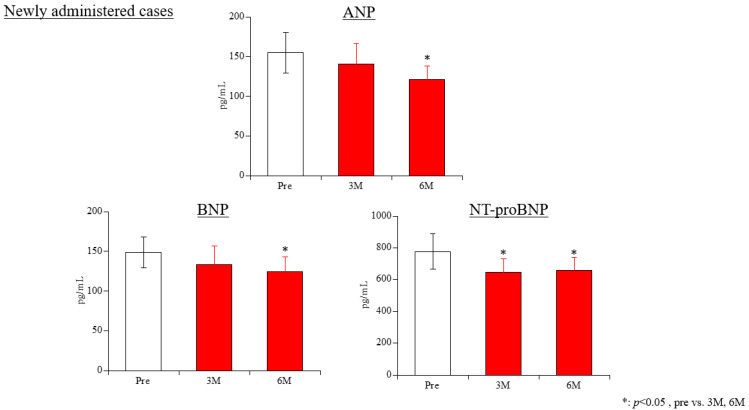
Changes in cardiac hormones in newly administered cases. ANP, atrial natriuretic peptide; BNP, brain natriuretic peptide; NT-proBNP, N-terminal pro-brain natriuretic peptide.

Cardiac-related biomarkers (ANP, BNP, NT-proBNP) (Figure 2): ANP levels were 154.8 ± 25.8 pg/mL at baseline, 140.8 ± 25.3 pg/mL at 3 months, and 121.3 ± 16.9 pg/mL at 6 months. A significant decrease was observed at 6 months compared to baseline (*p* = 0.018), but there was no significant difference between 3 and 6 months (*p* = 0.137). BNP levels were 148.7 ± 19.2 pg/mL at baseline, 133.4 ± 23.1 pg/mL at 3 months, and 124.4 ± 18.8 pg/mL at 6 months. A statistically significant decrease was observed at 6 months compared to baseline (*p* = 0.022), with no significant difference between 3 and 6 months (*p* = 0.491). NT-proBNP levels were 777.4 ± 109.8 pg/mL at baseline, 645.3 ± 85.1 pg/mL at 3 months, and 658.2 ± 82.8 pg/mL at 6 months. Statistically significant decreases were observed at both 3 months (*p* = 0.025) and 6 months (*p* = 0.046) compared to baseline, but no significant difference was found between 3 and 6 months (*p* = 0.475).

Renal-related biomarkers (BUN, Cr, eGFR) (Table 2): There was no significant change in BUN before and after treatment, nor across treatment time points. Serum creatinine (Cr) significantly decreased at 6 months compared to baseline (*p* = 0.023), although there were no significant differences between treatment periods. eGFR showed no significant change before and after treatment, or across time points.

Hepatic-related biomarkers (AST, ALT) (Table 2): No statistically significant changes were observed in AST or ALT levels before and after ferric citrate hydrate administration, or between treatment periods.

Other biomarkers (hs-CRP, Ox-LDL) (Table 2): There were no significant changes in hs-CRP levels before and after treatment, nor between treatment periods. Oxidized LDL (Ox-LDL) levels were significantly reduced at 3 and 6 months compared to baseline (3 months: *p* = 0.025; 6 months: *p* = 0.001), with no significant difference between 3 and 6 months (*p* = 0.164).

- <Switching cases>
- Primary endpoint:

Hb (Figure 1): In patients who switched from iron sulfate sustained-release tablets to ferric citrate hydrate, the hemoglobin level was 11.9 ± 1.4 g/dL before switching. After switching, the hemoglobin level increased significantly to 12.2 ± 1.2 g/dL at 1 month, 12.3 ± 1.2 g/dL at 3 months, and 12.4 ± 1.4 g/dL at 6 months (1 month: *p* < 0.001; 3 months: *p* = 0.009; 6 months: *p* = 0.004). There were no significant differences between post-switch time points (1 vs. 3 months: *p* = 0.503; 1 vs. 6 months: *p* = 0.156; 3 vs. 6 months: *p* = 0.214).

- Secondary endpoints:

NYHA classification (Table 3): No patients experienced worsening of NYHA classification after switching to ferric citrate hydrate. Compared to baseline, NYHA classification improved significantly (*p* = 0.044).

Ht, Fe, TSAT, and ferritin (Table 3): After switching, hematocrit values increased significantly compared to pre-switch levels (1 month: *p* = 0.003; 3 months: *p* = 0.012; 6 months: *p* = 0.020). However, there were no significant differences between the post-switch time points (1 vs. 3 months: *p* = 0.366; 1 vs. 6 months: *p* = 0.052; 3 vs. 6 months: *p* = 0.108). Serum iron levels did not differ significantly before and after switching, nor among the post-switch time points. Ferritin levels increased significantly from pre-switch to 1 month post-switch (*p* = 0.002), but no significant changes were observed between 3 and 6 months or among time points thereafter. TSAT levels increased significantly at 1 and 3 months post-switch (both *p* < 0.001). Additionally, TSAT continued to rise significantly at 3 and 6 months compared to 1 month (1 vs. 3 months: *p* = 0.009; 1 vs. 6 months: *p* < 0.001), and at 6 months compared to 3 months (*p* = 0.003).

**Figure 3:**
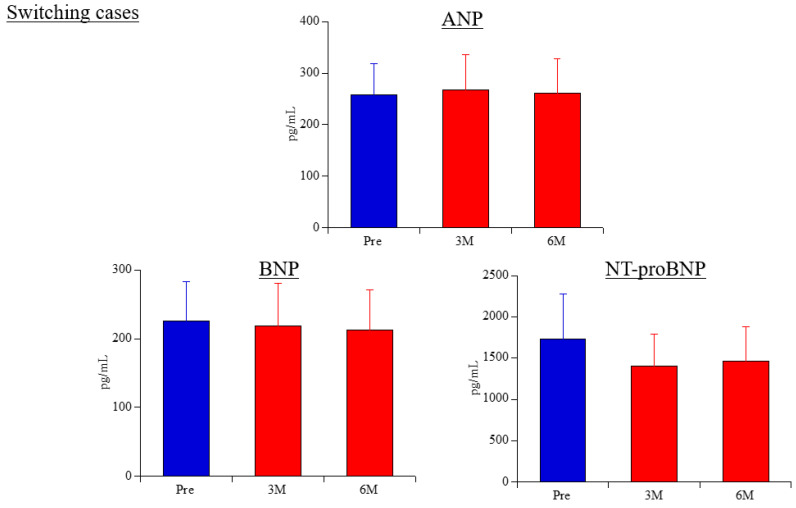
Changes in cardiac hormones in switching cases. ANP, atrial natriuretic peptide; BNP, brain natriuretic peptide; NT-proBNP, N-terminal pro-brain natriuretic peptide.

Cardiac-related biomarkers (ANP, BNP, NT-proBNP) (Figure 3): ANP levels were 258.4 ± 60.2 pg/mL before switching, 267.1 ± 69.2 pg/mL at 3 months post-switch, and 261.2 ± 66.1 pg/mL at 6 months. There were no statistically significant changes before and after switching, nor among the post-switch time points. BNP levels were 225.4 ± 58.2 pg/mL before switching, 218.2 ± 62.9 pg/mL at 3 months, and 212.2 ± 58.7 pg/mL at 6 months. No statistically significant differences were found. NT-proBNP levels were 1727.9 ± 553.0 pg/mL before switching, 1408.2 ± 384.0 pg/mL at 3 months, and 1459.2 ± 421.5 pg/mL at 6 months. There were no statistically significant changes observed before and after switching or between post-switch time points.

Renal-related biomarkers (BUN, Cr, eGFR) (Table 3): There were no statistically significant changes in BUN, serum creatinine (Cr), or estimated glomerular filtration rate (eGFR) before and after switching to ferric citrate hydrate, nor between post-switch time points.

Hepatic-related biomarkers (AST, ALT) (Table 3): No significant differences were observed in AST or ALT levels before and after treatment, or across post-switch time points.

Other biomarkers (hs-CRP, Ox-LDL) (Table 3): No significant changes were observed in hs-CRP levels before and after switching, or between the 3- and 6-month post-switch periods. Similarly, no statistically significant differences were observed in Ox-LDL levels before and after switching, or between the post-switch time points.

## 4. Discussion

Ferric citrate hydrate, the formulation used in this study, was approved in 2014 as a drug for treating hyperphosphatemia in chronic kidney disease and in 2021 as a drug for iron deficiency anemia. It is a drug that suppresses phosphorus absorption by combining with phosphate ions derived from food in the digestive tract to form a poorly soluble precipitate. Although there have been clinical study reports on ferric citrate hydrate in relation to hyperphosphatemia or iron deficiency anemia [17,18,19], there have been no reports of studies on heart failure with iron deficiency anemia. The results of this study showed that ferric citrate hydrate not only improves anemia but also improves iron metabolism and the heart failure biomarkers ANP, BNP, and NT-proBNP in patients with chronic heart failure complicated by iron deficiency anemia. Furthermore, improvement in NYHA classification was observed. In addition, there was no worsening of heart failure during treatment with ferric citrate hydrate, and it was thought to be a safe drug that could be used. In a switching study from iron sulfate sustained-release tablets, an increase in Hb was observed after switching to ferric citrate hydrate. There was no difference in serum iron levels before and after switching, but ferritin levels increased significantly after switching, and TSAT increased significantly after 1 month and 3 months of switching. Although there were no differences in ANP, BNP, or NT-proBNP, biomarkers for heart failure, before and after switching, NYHA classification was improved. These results suggest the following: (1) in patients with heart failure complicated by anemia, shortness of breath may have been due in part to anemia, and improvement in anemia led to relief of symptoms; (2) improvement in anemia may have contributed to symptom relief in heart failure, which could have subsequently led to improvements in ANP, BNP, or NT-proBNP levels.

According to WHO criteria, anemia is defined as a hemoglobin level of less than 13 g/dL for men and less than 12 g/dL for women, and in most previous clinical studies, WHO criteria have been used. In the present study, the authors examined lowering these hemoglobin levels and considered that the hemoglobin levels that should be treated clinically for anemia were less than 11 g/dL for men and less than 10.5 g/dL for women. They believe that this may have resulted in the improvement of each dataset.

Many clinical studies have been conducted on administering iron supplements to patients with chronic heart failure. In a report on oral iron supplements, the IRONOUT HF study, which targeted 225 HFrEF patients with iron deficiency, found no significant difference between the oral iron supplement group and the placebo group in terms of peak VO2 (*p* = 0.46), 6-min walk distance (*p* = 0.19), NT-proBNP levels (*p* = 0.48), or KCCQ clinical scores (*p* = 0.57) [16]. The reasons why no difference was seen were: (1) the mean Hb level before iron administration in the target patients was high at 12.6 (11.8–13.3) g/dL, (2) the mean NT-proBNP level before iron administration in the target patients was low at 1111 (453–2412) pg/mL compared to our study, (3) the TSAT level only increased by 2 (−3 to 7) % after iron treatment, (4) ferritin levels did not increase significantly after treatment with iron supplements, (5) the observation period was short (16 weeks), (6) adverse events were reported in 35% of cases, serious adverse events in 109% of cases, and permanent discontinuation of treatment in 14% of cases, and (7) it was stated that 113 out of 114 subjects received at least one dose of the study drug, so it was thought that the doses may not have been administered in sufficient quantities every day. Our research results have demonstrated that, in patients with heart failure, it is important to improve anemia as well as to supplement iron, and although there are many clinical studies on iron deficiency regardless of the presence or absence of anemia, there are few studies on iron deficiency anemia, so we believe that the results of this study are significant.

On the other hand, there are reports of the effectiveness of intravenous iron preparations, and the ESC guidelines also recommend intravenous iron supplementation for HFrEF and HFmrEF [15]. In the CONFIRM-HF study (observation period: 52 weeks), which included 304 patients with chronic heart failure and iron deficiency, patients in the intravenous iron group were given one or two vials (500 mg or 1000 mg of iron) at Day 0 and Weeks 6, 12, 24, and 36, depending on the patient’s weight and hemoglobin level. The results showed that the iron supplementation group had significantly better results than the placebo group in terms of the Patient Global Assessment, NYHA functional classification, 6-min walk test, fatigue score, and quality of life (KCCQ, EQ-5D). In addition, there was a 61% reduction in hospitalizations due to worsening heart failure compared to the placebo group (*p* = 0.009) [14]. In the FAIR-HF study (observation period: 24 months), which targeted 459 patients with chronic heart failure (NYHA II, III, HFrEF) with Hb 9.5 to 13.5 g/dL + iron deficiency, the iron supplementation group had significantly better results than the placebo group in terms of Patient Global Assessment (*p* < 0.001), NYHA classification (*p* < 0.001), and 6-min walking distance (*p* < 0.001) [13]. In the AFFIRM-AHF study (observation period: 52 weeks), which targeted 1110 patients with acute heart failure and iron deficiency (EF < 50%), the primary endpoint events (heart failure hospitalization, cardiovascular death) were reduced in the intravenous iron group (*p* = 0.059), and heart failure hospitalization was significantly reduced in the intravenous iron group (*p* = 0.013). In patients with heart failure, regardless of whether they have anemia or not, intravenous iron supplementation is effective [20]. In the IRONMAN trial (mean observation period: 2.5 years) of 1137 patients with chronic heart failure with EF < 45% who had iron deficiency, the impact of the COVID-19 pandemic meant that the trial was unable to show a significant difference in the primary endpoint (recurrent hospitalization for heart failure and cardiovascular death) (*p* = 0.07), but numerically, hospitalization and cardiovascular death decreased, and the quality of life of the participants in the intervention group improved significantly after 4 months [21]. The high iron content of the intravenous iron and the fact that it could be administered appropriately in the hospital meant that it was possible to provide sufficient iron supplementation, which may have contributed to the positive results. The author also believes that one of the reasons why large-scale studies have not shown the effectiveness of oral iron preparations is that oral iron preparations often cause gastrointestinal symptoms as side effects, which can make it difficult to continue taking them.

In our study, none of the patients who were newly introduced to ferric citrate hydrate had to discontinue taking it due to gastrointestinal symptoms. In our study, none of the patients who were newly introduced to Ferric Citrate Hydrate discontinued treatment due to gastrointestinal symptoms. Among the patients who switched to Ferric Citrate Hydrate, two experienced issues, but only one ultimately discontinued treatment. Furthermore, in the new cases, seven patients had previously taken iron sulfate sustained-release tablets and had been unable to continue taking them due to gastrointestinal symptoms. All seven of these patients were able to continue taking ferric citrate hydrate, indicating that its impact on gastrointestinal symptoms was minimal. Iron supplementation and anemia treatment could be carried out appropriately and continuously. The problem with conventional oral iron preparations is that they cannot be continued due to side effects such as gastrointestinal symptoms [22,23,24]. Ferric citrate hydrate, which was examined in this study, has been reported to cause fewer gastrointestinal complications than conventional iron preparations. A clinical study was conducted on 739 Japanese patients with iron deficiency anemia and no heart failure, and a comparison study was conducted on ferric citrate hydrate and the oral drug sodium ferrous citrate. In this study, ferric citrate hydrate was divided into high-dose (1000 mg/day) and low-dose (500 mg/day) and compared with sodium ferrous citrate (100 mg/day). Most of the adverse events that led to early discontinuation of the study treatment were related to gastrointestinal symptoms, and there was no difference in the incidence of diarrhea among the three groups. However, the incidence of nausea was 15.5% in the low-dose group, 10.5% in the high-dose group, and 32.7% in the sodium ferrous citrate group, and the incidence of vomiting was 5.2% in the low-dose group, 1.2% in the high-dose group, and 15.2% in the sodium ferrous citrate group. The incidence of these events was significantly lower in the ferric citrate hydrate group than in the sodium ferrous citrate group [25]. In terms of economic evaluation, it has been reported that switching to ferric citrate hydrate is more cost-effective when gastrointestinal side effects are observed with sodium ferrous citrate [26].

The signal pathways that induce nausea and vomiting remain unclear, but it has been suggested that serotonin and free radicals are involved in nausea and vomiting [27,28]. Machida et al. hypothesized that, since ferrous iron is more likely to produce free radicals and cause gastrointestinal disorders than ferric iron, the difference in the incidence of vomiting between sodium ferrous citrate and ferric citrate hydrate is due to the effect of iron supplements on small intestinal EC cell counts. They conducted a rat study to investigate effects of sodium ferrous citrate and ferric citrate hydrate. The sodium ferrous citrate group resulted in a significant increase in the number of EC cells in the duodenum and jejunum and the associated expression of substance, whereas the ferric citrate hydrate group did not demonstrate such effects. In addition, the sodium ferrous citrate group showed a significant decrease in food intake that continued from 48 h after the start of administration, while the ferric citrate hydrate group did not show such an effect [29]. With conventional oral iron preparations, there are many cases where patients are unable to continue taking the medication due to gastrointestinal symptoms, and this is one of the reasons why they do not produce the same results as intravenous iron preparations. The author believes that if the medication can be taken continuously and appropriately, it is possible to achieve the same results as intravenous medication, and based on our research results, ferric citrate hydrate produced very few gastrointestinal symptoms. In addition, the fact that all patients were able to continue taking the medication throughout the study period is also thought to be a reason for the good results.

In this study, 25% of newly administered cases and 35% of switching cases were taking HIF-PH inhibitors, which are drugs used to treat renal anemia, prior to the study. As many of the patients in this study were elderly, it was thought that renal anemia and iron deficiency anemia were relatively common in this group. Furthermore, it is said that HIF-PH inhibitors lower hepcidin and increase iron metabolism (iron utilization), and it is possible that the administration of HIF-PH inhibitors caused the decrease in ferritin and TSAT levels. Decreases in hepcidin, ferritin, and TSAT levels have been reported with HIF-PH inhibitors [30,31], and in some cases, the administration of iron supplements is necessary. It has also been reported that there are differences depending on the type of HIF-PH inhibitor [32,33]. In the results of our clinical study, in which we switched from ESA to four types of HIF-PH inhibitors, there was variation in the TSAT and ferritin data, and no difference in iron metabolism was observed between the types of HIF-PH inhibitors [34].

There is no fixed view on this. This point should be studied in the future, and the necessity of iron supplements should be clarified. The subjects in this study were patients with stable iron metabolism whose doses of HIF-PH inhibitors had remained unchanged for at least six months. As the next step, we plan to conduct a sub-analysis comparing patients with and without HIF-PH inhibitor treatment. Furthermore, we consider it necessary to investigate the potential effects of HIF-PH inhibitors on iron metabolism and heart failure.

## 5. Conclusions

This study found that ferric citrate hydrate significantly increased Hb, serum iron, TSAT, and ferritin levels in patients with chronic heart failure complicated by iron deficiency anemia, and decreased ANP, BNP, and NT-proBNP levels. Ferric citrate hydrate could be continued without side effects such as gastrointestinal symptoms. In a study comparing ferric citrate hydrate with iron sulfate sustained-release tablets, ferric citrate hydrate had better results for ferritin and TSAT, but there was no difference in Hb or serum iron levels. There was also no difference in ANP, BNP, or NT-proBNP.

We believe that improvement in iron metabolism and anemia due to iron supplementation led to improvements in heart failure biomarkers.

## 6. Limitations

There are several limitations to this study. First, this was a single-center, observational study evaluating the effects of ferric citrate hydrate, and not a randomized controlled trial with a placebo or alternative iron preparations. Although ferric citrate hydrate did not lead to worsening heart failure or re-hospitalization, the primary limitation is that the study endpoints were limited to biomarkers, without detailed assessments of quality of life (QOL) or exercise capacity. Including these parameters in future studies would allow for a more comprehensive evaluation of the efficacy of oral iron supplementation.

Second, increasing the sample size in future studies would help to better demonstrate the effectiveness of oral iron preparations in patients with chronic heart failure complicated by iron deficiency anemia. A comparative study including a control group would also be warranted.

Third, although the observation period in this study was 6 months, a longer-term follow-up would provide more clinically relevant data. Finally, for the treatment of heart failure, medications such as ARNIs and SGLT2 inhibitors may influence long-term outcomes. While the current study only included patients whose pharmacologic treatment had remained unchanged for at least 6 months, the specific effects of each drug should be investigated in future studies.

## Figures and Tables

**Table 1:** Patient characteristics.

	Newly Administered Cases	Switching Cases
Number	95	46
Age (years)	74.7 ± 1.2 (43–97)	77.4 ± 1.6 (47–92)
Gender (male:female)	64:31	29:17
Duration of Heart Failure (months)	11.6 ± 3.6	14.9 ± 5.7
NYHA classification	1.87 ± 0.65	
I	11	13
II	62	26
III	22	7
Average	2.12 ± 0.58	1.87 ± 0.65
Classifications of heart failure		
HFrEF	24 (25%)	16 (35%)
HFmrEF	20 (21%)	9 (19%)
HFpEF	51 (54%)	21 (46%)
Causes of heart failure		
Ischemic heart disease	25 (26%)	14 (30%)
Valve disease	49 (52%)	19 (41%)
Cardiomyopathy	4 (4%)	7 (15%)
Arrhythmia	4 (4%)	1 (2%)
Hypertension	12 (13%)	5 (11%)
Amyloidosis	1 (1%)	0 (0%)
Complication		
Hypertension	58 (61%)	25 (54%)
Diabetes mellitus	30 (32%)	20 (43%)
Dyslipidemia	68 (72%)	35 (76%)
Hyperuricemia	34 (36%)	21 (46%)
CKD (stage G3a>)	48 (51%)	26 (57%)
CKD (stage G3b>)	40 (42%)	24 (52%)
CKD (stage G4>)	12 (13%)	8 (17%)
Obesity	21 (22%)	7 (15%)
Cerebral infarct	3 (3%)	2 (4%)
Peripheral atrial disease	5 (5%)	4 (9%)
Treatment for heart failure		
ACE-I or ARB	31 (33%)	11 (24%)
ARNI	39 (41%)	23 (50%)
MRA	64 (67%)	30 (65%)
Beta-blockers	77 (81%)	39 (85%)
SGLT2 inhibitor	33 (35%)	17 (37%)
Ivabradine	2 (2%)	2 (4%)
Loop diuretics	40 (42%)	24 (52%)
Tolvaptan	11 (12%)	4 (9%)
Calcium channel blocker	31 (33%)	14 (30%)
Digoxin	3 (3%)	1 (2%)
Pimobendan	2 (2%)	2 (4%)
HIF-PH inhibitor use	24 (25%)	16 (35%)

NYHA, New York Heart Association; HFrEF, Heart Failure with Reduced Ejection Fraction; HFmrEF, Heart Failure with mildly reduced Ejection Fraction; HFpEF, Heart Failure with preserved Ejection Fraction; CKD, chronic kidney disease; ACE-I, Angiotensin-Converting Enzyme Inhibitor; ARB, Angiotensin II Receptor Blocker; ARNI, Angiotensin Receptor Neprilysin Inhibitor; MRA, Mineralocorticoid Receptor Antagonist; SGLT2, Sodium-Glucose Cotransporter 2 Inhibitor; HIF-PH, hypoxia-inducible factor prolyl-hydroxylase.

**Table 2:** Changes in New York Heart Association classification and each biomarker in the newly administered cases.

Newly Administered Cases	Pre	1 Month	3 Months	6 Months
NYHA classification	2.12 ± 0.58	-	-	1.74 ± 0.53
*p* value	-	-	-	<0.001
Hematocrit (%)	32.8 ± 0.3	37.8 ± 0.4	39.9 ± 0.5	39.6 ± 0.5
*p* value	-	<0.001	<0.001	<0.001
Serum iron (μg/dL)	47.5 ± 2.9	95.5 ± 6.2	84.6 ± 3.8	94.6 ± 3.7
*p* value	-	<0.001	<0.001	<0.001
TSAT (%)	15.1 ± 1.1	33.0 ± 2.3	29.8 ± 1.5	32.9 ± 1.4
*p* value	-	<0.001	<0.001	<0.001
Ferritin (ng/mL)	39.5 ± 6.4	66.3 ± 12.0	80.4 ± 11.5	114.9 ± 16.2
*p* value	-	<0.001	<0.001	<0.001
BUN (mg/dL)	20.5 ± 0.8	20.3 ± 0.7	20.0 ± 0.8	20.6 ± 0.8
*p* value	-	0.638	0.381	0.895
Serum creatine (mg/dL)	1.14 ± 0.05	1.13 ± 0.05	1.12 ± 0.05	1.11 ± 0.05
*p* value	-	0.377	0.226	0.023
eGFR (mL/dL/1.73 m^2^)	51.2 ± 1.8	50.8 ± 1.7	51.0 ± 1.8	51.6 ± 1.7
*p* value	-	0.403	0.787	0.518
AST (U/L)	24.2 ± 1.1	24.6 ± 0.9	24.9 ± 1.1	25.8 ± 1.1
*p* value	-	0.545	0.480	0.156
ALT (U/L)	16.3 ± 0.9	17.1 ± 0.8	17.5 ± 1.0	17.6 ± 0.9
*p* value	-	0.244	0.253	0.108
hs-CRP (mg/dL)	0.50 ± 0.11	0.38 ± 0.10	0.50 ± 0.10	0.33 ± 0.07
*p* value	-	0.088	0.959	0.092
Oxidized LDL (U/L)	57.6 ± 2.0	-	53.5 ± 2.0	51.3 ± 1.9
*p* value	-	-	0.025	0.001

NYHA, New York Heart Association; TSAT, transferrin saturation index; BUN, urea nitrogen; eGFR, estimated glomerular filtration rate; AST, aspartate aminotransferase; ALT, alanine aminotransferase; hs-CRP, high-sensitivity C-reactive protein; oxidized LDL, Oxidized low-density lipoprotein.

**Table 3:** Changes in New York Heart Association classification and each biomarker in the switching cases.

Switching Cases	Pre	1 Month	3 Months	6 Months
NYHA classification	1.87 ± 0.65	-	-	1.78 ± 0.59
*p* value	-	-	-	0.044
Hematocrit (%)	36.7 ± 0.7	37.8 ± 0.6	38.2 ± 0.6	38.7 ± 0.6
*p* value	-	0.003	0.012	0.02
Serum iron (μg/dL)	82.0 ± 4.8	95.2 ± 6.8	83.8 ± 4.1	89.8 ± 6.6
*p* value	-	0.117	0.753	0.327
TSAT (%)	28.9 ± 1.8	48.8 ± 4.1	42.6 ± 2.9	32.8 ± 1.9
*p* value	-	<0.001	<0.001	0.125
Ferritin (ng/mL)	192.4 ± 33.8	208.7 ± 36.2	197.8 ± 30.7	206.5 ± 30.5
*p* value	-	0.002	0.673	0.273
BUN (mg/dL)	25.4 ± 1.5	25.1 ± 1.2	24.5 ± 1.2	24.7 ± 1.5
*p* value	-	0.748	0.199	0.440
Serum creatine (mg/dL)	1.26 ± 0.10	1.25 ± 0.08	1.22 ± 0.08	1.23 ± 0.08
*p* value	-	0.753	0.289	0.498
eGFR (mL/dL/1.73 m^2^)	47.3 ± 2.9	46.0 ± 2.8	47.2 ± 2.8	46.5 ± 2.7
*p* value	-	0.090	0.905	0.471
AST (U/L)	25.7 ± 2.0	26.3 ± 1.8	24.8 ± 1.7	27.3 ± 2.9
*p* value	-	0.493	0.299	0.374
ALT (U/L)	18.5 ± 2.2	18.6 ± 1.8	17.5 ± 1.7	20.3 ± 2.8
*p* value	-	0.906	0.240	0.223
hs-CRP (mg/dL)	0.33 ± 0.06	0.25 ± 0.05	0.34 ± 0.06	0.43 ± 0.15
*p* value	-	0.113	0.842	0.388
Oxidized LDL (U/L)	56.2 ± 2.9	-	51.3 ± 2.5	53.9 ± 2.5
*p* value	-	-	0.050	0.425

NYHA, New York Heart Association; TSAT, transferrin saturation index; BUN, urea nitrogen; eGFR, estimated glomerular filtration rate; AST, aspartate aminotransferase; ALT, alanine aminotransferase; hs-CRP, high-sensitivity C-reactive protein; Oxidized LDL, oxidized low-density lipoprotein.

## Data Availability

The data that support the findings of this study are available on request from the corresponding author, [A.S]. The data are not publicly available due to restrictions e.g., their containing information that could compromise the privacy of research participants.
